# Hyperoxia causes senescence and increases glycolysis in cultured lung epithelial cells

**DOI:** 10.14814/phy2.14839

**Published:** 2021-05-27

**Authors:** Alejandro M. Scaffa, Abigail L. Peterson, Jennifer F. Carr, David Garcia, Hongwei Yao, Phyllis A. Dennery

**Affiliations:** ^1^ Department of Molecular Pharmacology and Physiology Brown University Providence Rhode Island USA; ^2^ Department of Molecular Biology, Cell Biology, and Biochemistry Brown University Providence Rhode Island USA; ^3^ Department of Chemistry Brown University Providence Rhode Island USA; ^4^ Department of Pediatrics Warren Alpert Medical School of Brown University Providence Rhode Island USA

**Keywords:** epithelial, glycolysis, hyperoxia, lung, metabolism, senescence

## Abstract

Supplemental oxygen and mechanical ventilation commonly used in premature infants may lead to chronic lung disease of prematurity, which is characterized by arrested alveolar development and dysmorphic vascular development. Hyperoxia is also known to dysregulate p53, senescence, and metabolism. However, whether these changes in p53, senescence, and metabolism are intertwined in response to hyperoxia is still unknown. Given that the lung epithelium is the first cell to encounter ambient oxygen during a hyperoxic exposure, we used mouse lung epithelial cells (MLE‐12), surfactant protein expressing type II cells, to explore whether hyperoxic exposure alters senescence and glycolysis. We measured glycolytic rate using a Seahorse Bioanalyzer assay and senescence using a senescence‐associated β galactosidase activity assay with X‐gal and C_12_FDG as substrates. We found that hyperoxic exposure caused senescence and increased glycolysis as well as reduced proliferation. This was associated with increased double stranded DNA damage, p53 phosphorylation and nuclear localization. Furthermore, hyperoxia‐induced senescence was p53‐dependent, but not pRB‐dependent, as shown in p53KO and pRBKO cell lines. Despite the inhibitory effects of p53 on glycolysis, we observed that glycolysis was upregulated in hyperoxia‐exposed MLE‐12 cells. This was attributable to a subpopulation of highly glycolytic senescent cells detected by C_12_FDG sorting. Nevertheless, inhibition of glycolysis did not prevent hyperoxia‐induced senescence. Therapeutic strategies modulating p53 and glycolysis may be useful to mitigate the detrimental consequences of hyperoxia in the neonatal lung.

## Graphical abstract

Hyperoxia‐induced senescence is p53‐dependent, but not pRB‐dependent in MLE‐12 cells. Glycolysis was upregulated in hyperoxia‐exposed MLE‐12 cells, this increased glycolysis is derived from highly glycolytic senescent cells.

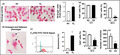

## 1 INTRODUCTION

Due to medical advances, premature infants born as early as 22 weeks of gestation can survive. However, their lungs are still in the canalicular and saccular stages of lung development and are unable to diffuse oxygen (Thébaud et al., 2019). As a life‐saving measure, premature infants are provided with supplemental oxygen (hyperoxia). This leads to sequelae since many infants develop chronic lung disease (CLD) of prematurity, a disorder characterized by arrested alveolar development, inflammation, and damaged alveoli (Hilgendorff & O'Reilly, 2015).

Hyperoxia causes damage, in part by generating reactive oxygen species (ROS) in the lung, leading to increased DNA damage (Barker et al., 2006). In mice, hyperoxia produces a phenotype similar to CLD (Berger & Bhandari, 2014). In cultured cells, hyperoxia leads to increased cell death and decreased cellular proliferation (Mantell et al., 1999). Hyperoxia is known to dysregulate lipid and tricarboxylic acid cycle metabolism (Dennery et al., 2018), senescence in vitro (Klimova et al., 2009), and also alters p53 signaling (O'Reilly et al., 1998). In addition, prior research using fibroblasts and bone osteosarcoma epithelial cells suggests that metabolism and replicative senescence are interdependent (Jiang et al., 2013), however, whether metabolism alters senescence or vice versa is not understood in hyperoxia‐exposed lung cells.

Senescence is a protective response to stress in which cells acquire a non‐proliferative state and develop a detrimental pro‐inflammatory senescence associated secretory phenotype (SASP) (Coppé et al., 2010). Senescence is often associated with aging (Childs et al., 2014), but it also plays beneficial roles in wound healing (Demaria et al., 2017). At the center of the senescence pathway is the tumor suppressor protein p53, which transcriptionally activates p21 and p16 leading to cyclin dependent kinase (CDK) inhibition, which increases pRB binding to E2F1, preventing its role as a transcription factor, leading to cell cycle arrest and senescence (Qian & Chen, 2013). There are several forms of senescence including replicative senescence, which is associated with aging and is usually mediated by p53 and p16 (Beauséjour et al., 2003). Developmentally induced senescence is regulated by p21 during fetal development. This allows for tissue remodeling and organ differentiation (Storer et al., 2013). In contrast, stress‐induced senescence is acquired after damage to double‐stranded DNA through ROS. This type of senescence is usually mediated by p53 and pRB, but is also stress‐specific (Demaria et al., 2017; Le et al., 2010). Whether senescence plays a protective or detrimental role is not fully understood, and it is often specific to the stress that generates it.

Metabolic dysfunction has been implicated in lung injury and repair in neonatal models of hyperoxic exposure (Dennery et al., 2018; Kurzner et al., 1988; La Frano et al., 2017). In fact, endothelial, epithelial, and fibroblast cells show metabolic dysregulation in response to hyperoxia (Boros et al., 2002; Das, 2013; Yao et al., 2017). Senescent cells also show metabolic dysregulations, but it is not known whether this metabolic dysregulation leads to senescence or vice versa. While p53 is upregulated by hyperoxia (O'Reilly et al., 1998), and it plays a role in mediating senescence and modulating glycolysis (Bensaad et al., 2006; Itahana & Itahana, 2018; Kondoh et al., 2005; Mikawa et al., 2014; Puzio‐Kuter, 2011; Wang et al., 2014), it is unknown whether senescence and glycolysis are interdependent through p53 in hyperoxia‐exposed cells. If they were, this would provide strategies to modulate senescence induced by hyperoxia through alterations in metabolism. Type II (ATII) cells are progenitor to ATI cells, the alveolar cells that are responsible for gas exchange, and therefore provide an important regenerative function in response to stress in the lung (Nova et al., 2019; Olajuyin et al., 2019; Vaughan & Chapman, 2017). However, ATII cell numbers decrease in response to hyperoxia (O'Reilly et al., 1998), thereby preventing lung repair.

Previous reports showed that hyperoxia for 7 days induces senescence in fibroblasts (Parikh et al., 2019), and that 3 days of hyperoxia leads to senescence that requires p53 and pRb signaling in lung fibroblast cells (Klimova et al., 2009). Whether ATII cells exposed to 24 h of hyperoxia become senescent and whether this is also mediated by p53 and pRB remains to be determined. Given that hyperoxia upregulates senescence and can also upregulate p53 protein in mouse alveoli (O'Reilly et al., 1998), we hypothesized that hyperoxia‐induced senescence is p53‐dependent in ATII‐like mouse lung epithelial cells (MLE‐12). We further hypothesized that hyperoxia also leads p53‐mediated downregulation of glycolysis which may serve to counter senescence and its detrimental effects.

## 2 METHODS

### 2.1 MLE‐12 cell culture and hyperoxic exposure

MLE‐12 cells (ATCC) were grown in Dulbecco's medium:Ham's F12 (50:50 mix) with 2% FBS, 1% Pen/strep, 10 nM hydrocortisone, insulin‐transferrin‐selenium (0.005 mg/ml insulin, 0.01 mg/ml transferrin, 30 nM sodium selenite), and 10 nM β‐estradiol. Cells at 50% confluency were incubated at 37°C in air (21% O_2_ and 5% CO_2_) or hyperoxia (95% O_2_ and 5% CO_2_) for 4, 8, 12 or 24 h in a humidified chamber.

### 2.2 Transcriptional analysis

Quantitative real time PCR (RT‐qPCR) assays were performed using the ThermoFisher Taqman qPCR master mix and Taqman fluorescent probes. All qPCR assays were done with 18s (Taqman Hs99999) as a housekeeping control in an ABI5000 instrument.

Probes used were: Rb1 (Mm00485586), E2F1 (Mm00432939), MMP3 (Mm00440295), MMP10 (Mm01168399), p53 (Mm01731290), p16 (Mm00494449_m1), p21 (Mm00494449), HK2 (Mm00443385), Tp53 inducible glycolysis and apoptosis regulator (TIGAR) (Mm00621530), glucose transporter 1 protein (GLUT1) (Mm00449511), PFKFB3 (Mm00504650), and phosphofructokinase (PFKM) (Mm01309576).

### 2.3 Detection of cellular proteins

Assay performed as previously described (Mahmood & Yang, 2012). Protein levels were analyzed using the ChemiDoc Touch Imaging System (BioRad). Calnexin was used as loading control for all western blots.

Antibodies used were: Abcam: Anti‐H2AX phosphoS139 (ab26350), CRISPR‐Cas9 (ab204448), GLUT1 (ab115730), hexokinase II (HKII, ab131196), p16 (ab54210), p21 (ab107099), p53 (ab31333), phosphor‐p53 S15 (ab1431), anti‐p53 phospho‐S392 (ab33889), Tigar (ab62533). Bethyl Laboratories: H2AX (A300‐083A). Cell Signaling: p53 (9282), p53 Ser15 (9284), p53 Ser392 (9281). Enzo: calnexin (ADI‐SPA‐860‐F). Millipore: Gamma H2AX (05–636), phospho‐histone H2A Ser139 (05–636). Santa Cruz: β‐actin (sc‐1616).

### 2.4 Immunofluorescence

MLE‐12 cells were fixed in 5% formalin and stained as previously described (Donaldson, 2001). Primary antibodies included p53 (abcam 31333 at 1:200 dilution), phospho‐histone H2A Ser139 (Millipore 05‐636 at 1:100 dilution) and 53BP1 (Novus Biologicals NB100‐304 at 1:1000 dilution). Images were acquired using a Zeiss Axiovert 200 M Fluorescence Microscope at magnification 200×.

### 2.5 Phospho‐p53 measurement by ELISA

Lysates were collected with phosphatase inhibitors, and phosphor‐p53 was measured using a Cell Signaling (#7365) phosphorylated Ser15 p53 ELISA kit as per the manufacturer's instruction.

### 2.6 Glycolytic rate assay (GRA)

We seeded 22,000 MLE‐12 cells/well into a Seahorse XF24 microplate (Agilent) and exposed them to air or hyperoxia. This cell density was determined to be optimal for this cell line (data not shown). Basal glycolytic rate (referred to as glycolytic rate) and compensatory glycolytic rates were obtained and measured as previously published (Romero et al., 2017) and as per manufacturer's instructions. Injections included in the assay were rotenone/antimycin A (0.5 μM, final) and 2‐deoxy‐d‐glucose (2‐DG, 50 mM, final). Data were expressed as a ratio to cell numbers.

### 2.7 Detection of senescence

#### 2.7.1 SA‐β‐gal acivity

MLE‐12 cells were seeded in a six‐well plate fitted with glass slides and exposed to air or hyperoxia. Senescence‐associated β galactosidase (SA‐β‐gal) activity was measured at pH6.0 by cytochemistry using X‐gal as the substrate as previously described (Itahana et al., 2013). Images were acquired with a Zeiss Axiovert 200M Brightfield/Fluorescence Microscope at magnification 200×, using only the brightfield function.

#### 2.7.2 C_12_FDG assay and sorting

We seeded 400,000 MLE‐12 cells/well in a six‐well plate in triplicate. SA‐β‐gal activity was measured by FACS using C_12_FDG as a substrate as previously described (Debacq‐Chainiaux et al., 2009). Further analysis was performed with FlowJo (Oregon, FlowJo LLC). Sixty thousand hyperoxia‐exposed cells/well were then sorted onto Seahorse plates, based on C_12_FDG fluorescence as a measure of senescence.

### 2.8 Cell proliferation assay

Proliferation was assessed by Click‐iT^®^ EdU Flow Cytometry Assay Kit from Invitrogen according to the manufacturer's protocol. Briefly, cells were incubated with 10 μM of a nucleoside analog of thymidine (5‐ethynyl‐2′deoxyuridine, EdU) for 2 h. After washing and fixation, incorporated EdU during DNA synthesis was labeled with Alexa Fluor 488 azide in the provided reaction buffer for 30 min. Cells were analyzed using the BD FACSAria (BD Bioscience) and the percentage of EdU positive cells was calculated.

### 2.9 Colony‐forming unit assay

After exposure of MLE‐12 cells to air or hyperoxia for 24 h, 1×10^2^ cells were seeded in six‐well plates at 37°C in 21% O_2_ and 5% CO_2_, for 15 days. Thereafter, plates were incubated with 0.5% crystal violet (Sigma‐Aldrich) for 5 min at room temperature. Stained colonies with >50 cells were counted.

### 2.10 Quantification of p53 nuclear or cytoplasmic immunofluorescent signal

We quantitatively investigated p53 protein translocation to the nucleus by computational segmenting of cell nuclei in fluorescent microscopy images. We used adaptive thresholding algorithms to distinguish foreground from background and watershed algorithms to segment cell nuclei. Incorrect segmentations (multiple clumped nuclei) were found using advanced image processing methods (distance transform, graph cut) and removed from final results. The computer language used was the Python, System: Anaconda navigator, through a Brown University Server in the Wong Lab.

### 2.11 CRISPR/Cas9 knockout

A two‐step CRISPR system (Sigma‐Aldrich) was used as per the manufacturer's instructions. First, lentivirus containing Cas9 plasmid and blasticidin resistance was used to infect MLE‐12 cells. Blasticidin selection led to Cas9+ MLE‐12 cells, which were transduced with lentivirus containing blank, p53 (ACCATCGGAGCAGCGCTCATGG) or pRB (AGAAAGTTTCATCCGTGGATGG) puromycin+lentivirus guides. Cells were then selected with puromycin and sorted into 96‐well plates using FACS. Knock‐out efficiency was determined by Western blot.

### 2.12 Statistical Analysis

Prism 6 software (GraphPad Software) was used to conduct all statistical analyses. The results were expressed as mean ± SEM of 6 biological replicates. One‐way ANOVA with Bonferroni correction for post‐hoc multiple comparisons was performed to measure the effect of one factor. The statistical significance of the differences between groups was evaluated by using two‐way ANOVA for overall significance.

## 3 RESULTS

### 3.1 Hyperoxia causes senescence in MLE‐12 cells

**FIGURE 1:**
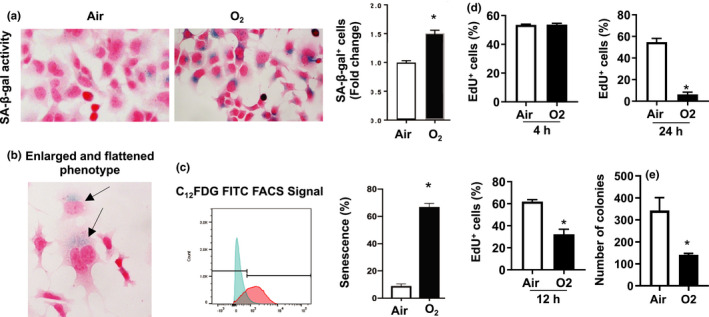
Hyperoxia causes senescence in mouse lung epithelial (MLE‐12) cells. MLE‐12 cells were exposed to 21% O_2_/5% CO_2_ (Air) or 95% O_2_/5% CO_2_ (O_2_) for 24 h. (a) Senescence levels in air and O_2_ were measured by the senescence associated beta galactosidase (SA‐β‐gal) activity, staining senescent cells blue. Nuclear fast red stains the nucleus red for counting purposes. (b) SA‐β‐gal assay representative image of senescent cells’ enlarged and flattened phenotype. (c) Senescence levels were quantified using the C_12_FDG FACS senescence assay. Emission spectrum is shown in blue for Air, and O_2_ is in red. (d) EdU incorporation was measured by flow cytometry in cells exposed to hyperoxia for 4, 12, and 24 h. (e) Number of colonies were counted in cells exposed to hyperoxia for 24 h followed by air recovery for 15 days. *p < 0.05 versus air groups. EdU, 5‐ethynyl‐2′deoxyuridine

SA‐β‐gal activity, as measured through the SA‐β‐Gal colorimetric assay is the hallmark of senescence (Dimri et al., 1995). SA‐β‐Gal activity is specifically measured at pH6 to exclude other β‐galactosidase activities. To measure senescence in live cells, the C_12_FDG assay is used, allowing for measurement of SA‐β‐gal activity through fluorescence signal acquisition (Debacq‐Chainiaux et al., 2009). Using SA‐β‐gal staining, we observed a 1.5‐fold increase in senescence after hyperoxia (Figure 1a). Cells exposed to hyperoxia showed an enlarged and flattened phenotype characteristic of senescence (Figure 1b). Using the C_12_FDG assay, we observed 5% senescence in the air compared to 65% in the hyperoxia group (Figure 1c). Hyperoxia for 12 h reduced EdU incorporation in MLE‐12 cells, and this was further decreased after 24 h of hyperoxic exposure (Figure 1d). In addition, the number of colonies seen in hyperoxia‐exposed MLE‐12 cells was significantly reduced from those in air‐exposed cells (Figure 1e). These results suggest that hyperoxia causes senescence and reduces proliferation in mouse lung epithelial cells.

### 3.2 Hyperoxia increases DNA damage and p53 activation

**FIGURE 2:**
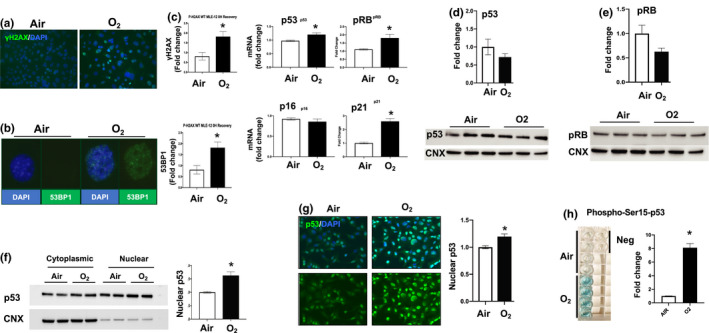
Hyperoxia causes DNA damage and p53 activation. Mouse lung epithelial (MLE‐12) cells were exposed to 21% O_2_/5% CO_2_ (Air) or 95% O_2_/5% CO_2_ (O_2_) for 24 h. For immunofluorescence, Blue = DAPI, nuclei; Green = protein of interest, Cyan = overlapping blue and green signal. (a) Measured γH2AX levels using immunofluorescence. (b) Measured 53BP1 levels using immunofluorescence, quantified by macros script. (c) Transcriptional levels of p53, pRB, p16, and p21 measured by RT‐qPCR. (d, e) p53 and pRB protein levels measured by western blot. Western blot normalized using calnexin (CNX) levels. (f) p53 nuclear and cytoplasmic levels measured by western blot from nuclear and cytoplasmic extracts. (g) Nuclear quantification of p53 using immunofluorescence. (h) Levels of phosphorylated p53 at Ser15 as measured by ELISA. Sample turns blue according to phosphorylated levels of p53 at Ser15, absorbance measured at 450 nm. *p < 0.05 versus air groups. RT‐qPCR, quantitative real time PCR

Since DNA damage can lead to senescence, we measured γH2AX signal intensity, which is a known marker of double stranded DNA damage. Using immunofluorescence, we found that hyperoxia‐exposed MLE‐12 cells had a 1.7‐fold increase in γH2AX protein levels compared to air controls as demonstrated by the number and intensity of γH2AX foci (Figure 2a). Another marker of double stranded DNA repair is 53BP1, which interacts with p53 and H2AX (Fernandez‐Vidal et al., 2017; Kleiner et al., 2015; Panier & Boulton, 2014). Immunoreactive 53BP1 signal was detected with immunofluorescence. Cells were counted as positive if they had one or more 53BP1 foci. Air controls had 0% 53BP1^+^ cells while hyperoxia‐exposed cells were 75% 53BP1^+^ (Figure 2b). We investigated transcript and protein levels of the core genes in the p53‐senescence signaling pathway in order to understand whether these contributed to hyperoxia‐induced senescence. Taqman assays revealed significant upregulation of p53, p21, and pRB mRNA in hyperoxia compared to air controls. In contrast, p16 mRNA levels were no different between hyperoxia‐exposed cells and air controls (Figure 2c). Hyperoxia did not change the total protein levels of p53 or pRB as seen by Western blots (Figure 2d,e). However, hyperoxia led to increased p53 nuclear translocation as indicated by Western blotting of nuclear/cytoplasmic fractions and by immunofluorescence (Figure 2f,g). The senescence proteins p21 and p16 could not be detected by Western blots.

Nuclear translocation of p53 can occur due to phosphorylation of serine15 (Melnikova et al., 2003), which has been shown to increase senescence and the DNA damage response (Qian & Chen, 2013). We used an ELISA assay to quantify phosphorylation of Ser15‐p53 (P‐Ser15‐p53) after hyperoxic exposure and found that P‐Ser15‐p53 was increased 8‐fold compared to air controls (Figure 2h). This suggests that hyperoxia induces phosphorylation of p53, facilitating its nuclear entry and downstream signaling roles.

### 3.3 Hyperoxia‐induced senescence is p53‐dependent but pRB‐independent

**FIGURE 3:**
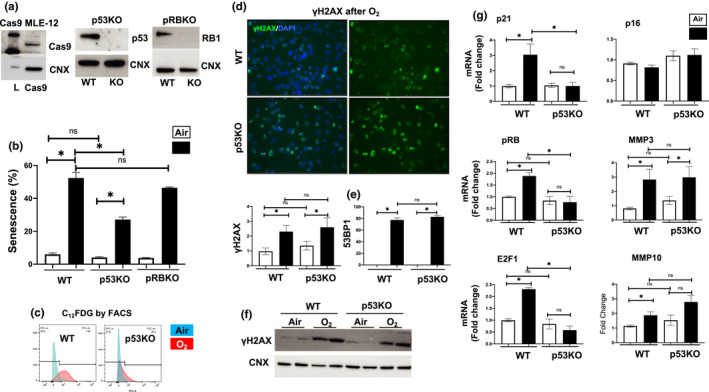
Hyperoxia‐induced senescence is p53‐dependent and pRB‐independent. (a) Cas9 knock‐in, p53KO, and pRBKO shown by Western blot, and calnexin (CNX) as a housekeeping control. (b) Senescence levels using C_12_FDG FACS of WT, pRBKO, and p53KO MLE‐12 cells exposed to 21% O_2_/5% CO_2_ (Air, white) or 95% O_2_/5% CO_2_ (O_2_, black) for 24 h. (c) Emission spectrum of senescent cells is shown in blue for Air, and O_2_ is in red. (d) γH2AX and (e) 53BP1 levels were measured using immunofluorescence. Blue = DAPI, nuclei; Green = protein of interest, Cyan = overlapping blue and green signal. (f) γH2AX protein levels were measured by Western blot in p53KO and WT cells exposed to hyperoxia. (g) Transcriptional levels of p21, pRB, E2F1, p16, MMP3, MMP10 measured by RT‐qPCR. *p < 0.05. MLE‐12, mouse lung epithelial

In order to understand whether p53 or pRB is necessary for the increased senescence observed in hyperoxia, we generated p53 and pRB knockout cell lines using CRISPR/Cas9 (Figure 3a). Using the C_12_FDG assay, we determined that air controls for WT, p53KO, and pRBKO cells had similar levels of senescence. However, hyperoxia‐exposed WT cells had 52.4% senescence compared to 27.1% senescence in the p53KO cells. (Figure 3a,b). This was corroborated by the spectrographic representation of the flow cytometry results (Figure 3c), where the p53KO hyperoxia samples showed a phenotype more like that of air‐exposed WT cells. In contrast, pRBKO showed no significant change in senescence levels in response to hyperoxia compared to similarly exposed WT (Figure 3b).

Western blotting and immunofluorescence assays confirmed that hyperoxia led to DNA damage, however, levels of γH2AX did not differ between WT and p53KO air‐exposed cells. Similarly, the WT hyperoxia‐exposed cells showed no difference in γH2AX compared to the p53KO hyperoxia‐exposed cells (Figure 3d,f). In addition, there were no differences in 53BP1 fluorescence between the two cell lines when exposed to air (Figure 3e). While the number of 53BP1 positive cells were similar between WT and p53KO hyperoxia‐exposed cells, there was an increased number of 53BP1 foci per cell in the p53KO compared to WT.

Transcriptional analysis of genes in the senescence pathway revealed that WT and p53KO air‐exposed cells had similar levels of p21, pRB, p16, or E2F1 mRNA. These genes were all upregulated in WT cells in response to hyperoxia whereas they were not in p53KO cells exposed to hyperoxia (Figure 3g). These data show that the senescence pathway is transcriptionally disrupted in p53KO cells and therefore p53 dependent. Hyperoxia increased the expression of the SASP genes MMP3 and MMP10 in WT cells (Figure 3g). This was not further increased in p53KO cells exposed to hyperoxia. Altogether, these data show that hyperoxia‐induced senescence is p53‐dependent.

### 3.4 Glycolysis is increased by hyperoxia but inhibited by p53

**FIGURE 4:**
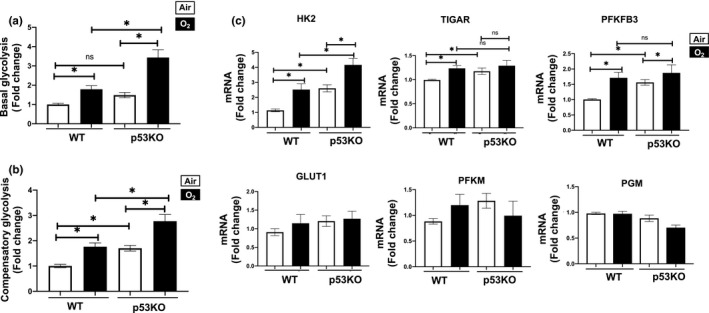
Hyperoxia leads to increased glycolysis while p53 is inhibiting glycolysis. Mouse lung epithelial (MLE‐12) cells were exposed to 21% O_2_/5% CO_2_ (Air, white) or 95% O_2_/5% CO_2_ (O_2_, black) for 24 h. Glycolysis rates were measured using the Seahorse Bioanalyzer Glycolytic Rate Assay (GRA). (a) Glycolysis and (b) compensatory glycolysis rates were measured using the Seahorse Bioanalyzer. Seahorse GRA used injections of Antimycin A + Rotenone, and 2‐DG for collection of various glycolytic parameters. (c) Transcriptional levels of HK2, GLUT1, TIGAR, PFKFB3, PGM, and PFKM were measured by RT‐qPCR. *p < 0.05. RT‐qPCR, quantitative real time PCR

Others have shown that glycolysis can be mediated by p53 (Bensaad et al., 2006; Itahana & Itahana, 2018; Liu et al., 2019; Zhang et al., 2013). Glycolysis rates observed with the GRA were 1.7‐fold higher in air‐exposed p53KO compared to air‐exposed WT. This suggests that loss of p53 increases glycolysis. After hyperoxia, glycolysis was upregulated in both cell lines. Nevertheless, the p53KO cells exposed to hyperoxia had a 2‐fold increase in glycolysis compared to similarly exposed WT cells. This same trend was observed with compensatory glycolysis (Figure 4a,b).

To further corroborate the effect of hyperoxia and p53 on glycolysis we measured genes regulating glycolysis. The glycolytic rate limiting enzyme HK2 showed a 2.2‐fold increase in air‐exposed p53KO compared to air‐exposed WT. After hyperoxia, HK2 mRNA levels were upregulated in both cell lines. The p53KO cells exposed to hyperoxia had a 1.7‐fold increase in HK2 transcription compared to WT cells that were similarly exposed (Figure 4c). The GLUT1 was unchanged in any condition. The TIGAR, which generates fructose 2, 6 biphosphate (F2,6BP) leading to inhibition PFKM, was upregulated in WT cells exposed to hyperoxia compared to air. However, there were no differences in p53KO air‐exposed cells compared to WT air, or between p53KO hyperoxia‐exposed cells and similarly exposed WT cells. Levels of phosphoglycerate mutase (PGM) mRNA, one of the rate‐limiting enzymes in glycolysis, showed no differences between air‐exposed WT and p53KO cells. However, there was a decrease in PGM in p53KO cells exposed to hyperoxia compared to WT cells similarly exposed. Lastly, PFKM mRNA levels were no different between p53KO and WT in air or hyperoxic exposures (Figure 4c). Overall, these data suggest that p53 inhibits glycolysis in WT cells, likely through the suppression of HK2. What upregulates HK2 in response to hyperoxia in p53KO cells is unknown and could explain the upregulated glycolysis observed in p53KO cells exposed to hyperoxia.

### 3.5 Changes in senescence and glycolysis are concurrent but are not interdependent

**FIGURE 5:**
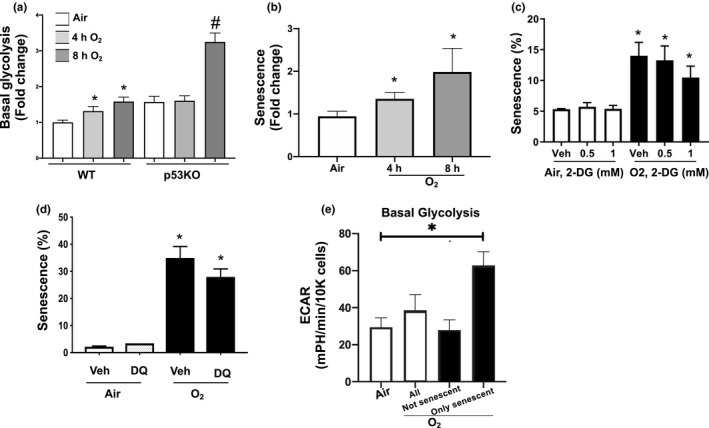
Hyperoxia‐induced senescent cells are highly glycolytic, but is not dependent on senescence. (a) Glycolysis rate was measured using the Seahorse Bioanalyzer's Glycolytic Rate Assay for WT and p53KO mouse lung epithelial (MLE‐12) cells exposed to 21% O_2_/5% CO_2_ (Air) or 95% O_2_/5% CO_2_ (O_2_) for 0–8 h in 4 h intervals. (b) Senescence levels in WT MLE‐12 cells exposed to O_2_ for 0, 4, and 8 h. (c) Senescence levels quantified using the C_12_FDG FACS senescence assay in response to varying concentrations of 2‐deoxy‐glucose (2‐DG), a glycolysis inhibitor. (d) Senescence levels quantified using the C_12_FDG FACS senescence assay in response to senolytics dosage 50 nM dasatinib plus 50 nM quercetin (DQ), the maximum dosage before cell death in air. (e) Glycolysis levels of sorted senescent cells using the C_12_FDG FACS senescence assay for senescence identification. Glycolysis measured Seahorse Bioanalyzer GRA. Categories: Air unselected cells, O_2_ unselected cells, O_2_ cells selected for cells with 50% least absorbance (less senescent), and 50% most absorbance (most senescent). *p < 0.05 compared to air or veh

In order to determine whether senescence and glycolysis are co‐regulated, we examined the timing of onset of glycolysis and senescence in MLE‐12 cells exposed to 4 and 8 h of hyperoxia. Glycolysis increased 2‐fold at 4 h in the WT cells exposed to hyperoxia compared to air controls. In contrast, in the p53KO, increases in glycolysis occurred only at 8 h of hyperoxia (Figure 5a). This suggests that p53 signaling accelerates the onset of glycolysis when cells are exposed to hyperoxia.

As to senescence, increased C_12_FDG hydroxylation was seen as early as 4 h after hyperoxia in the WT cells compared to air controls. In contrast, p53KO cells did not show a significant increase in senescence until 8 h of hyperoxia. This suggests that p53 slows the onset of senescence as well. The onset of senescence and glycolysis after short term hyperoxia in WT cells seem to occur concurrently. Since both the onset of senescence and glycolysis occurred at the same time, we could not demonstrate a causal relationship between the two phenomena (Figure 5a,b).

We then attempted to inhibit glycolysis in order to measure its effect on hyperoxia‐induced senescence. Using the Seahorse Bioanalyzer, we determined that 3 mM was the minimal concentration of 2‐DG needed to inhibit glycolysis by 80% but this concentration was toxic to cells. We tried various concentrations of 2‐DG, from 0.5 to 3 mM, but none led to a significant change in senescence in response to hyperoxia (Figure 5c).

In order to understand whether senescence causes changes in glycolysis, we sought to inhibit it with known senolytics dasatinib (D) and quercetin (Q) (Zhu et al., 2015). Varied concentrations of the two agents were used alone and in combination for 24 h without any effect on senescence levels in hyperoxia (Figure 5d). Higher doses than 50 nM were toxic to the cells. Therefore, we were not able to document whether changes in senescence affect glycolysis in MLE‐12 cells. Interestingly, quercetin is also an inhibitor of glucose transport and picomolar levels of quercetin alone led to increased cell proliferation in response to hyperoxia (data not shown).

To understand whether the senescent cells contributed to the increase in glycolysis, we sorted senescent cells identified with C_12_FDG, and seeded them into Seahorse plates for GRA analysis. Interestingly, the senescent cells had a 2.2‐fold increase in glycolysis compared to non‐senescent cells similarly exposed to hyperoxia (Figure 5e). This suggests that senescent cells contribute more to glycolysis than non‐senescent cells. This may explain the increased glycolysis observed after hyperoxic exposure.

## 4 DISCUSSION

Hyperoxia has been shown to dysregulate p53 (O'Reilly et al., 1998), senescence (Klimova et al., 2009; Parikh et al., 2019; You et al., 2019), and metabolism (Das, 2013; Dennery et al., 2018; Zhao et al., 2018). Others show that senescence is detrimental in most chronic lung disorders (Hamsanathan et al., 2019), yet the role of senescence in CLD remains largely unexplored. Specifically, there is a gap in knowledge as to whether hyperoxia leads to senescence in ATII cells. To our knowledge, this is the first study to show that hyperoxia exposure leads to senescence that is p53‐dependent in MLE‐12 cells, a type II like cell line. Senescent cells have dysregulated metabolism, and p53 plays a large role in glycolysis (Wiley & Campisi, 2016). Notably, metabolic dysregulation is a hallmark of the pathology of CLD as well (Dennery et al., 2018). Because of this, we also explored the relationship between senescence and glycolysis. Here we show that proliferation is inhibited when hyperoxia exposed MLE‐12 cells became senescent, and that this effect extends beyond the period of hyperoxic exposure. Furthermore, hyperoxia leads to increased glycolytic activity and senescent cells are highly glycolytic compared to similarly exposed cells that are not senescent.

Increased H2AX phosphorylation as well as increased 53BP1 expression and increased 8‐oxo‐2'‐deoxyguanosine staining (data not shown) suggest that hyperoxia caused DNA damage, despite the fact that hyperoxia‐induced oxidative stress itself could increase H2AX phosphorylation independent of DNA damage via ATM (Crowe et al., 2006). A key response to DNA damage is the upregulation of p53 (Lakin & Jackson, 1999; Williams & Schumacher, 2016). Indeed, we observed that p53 mRNA was upregulated immediately after hyperoxia. Even though overall p53 protein levels did not increase, nuclear localization and p53 phosphorylation increased in response to hyperoxia. The phosphorylation is likely mediated by ATM and ATR, which are known to phosphorylate p53 at Ser15, localizing it in the nucleus (Loughery et al., 2014). It is known that ATM and ATR are phosphorylated in response to DNA damage (Maréchal & Zou, 2013). When measuring senescence in the p53KO MLE‐12 cells, we observed that hyperoxia‐induced senescence was partially p53 dependent. It is unclear what signaling mechanisms account for the remaining senescence. It is unlikely that the senescence is due to p21 in our model given that p21 transcription was ablated in the p53KO cells. Although pRB is required for hyperoxia‐induced senescence in lung fibroblasts (Klimova et al., 2009), this was not the case in our study. Since p16 exerts its function by inhibiting CDK4‐mediated phosphorylation of pRB, hyperoxia‐induced senescence in lung epithelial cells is not dependent on the p16/pRB pathway. Indeed, not all forms of senescence activate p16 pathway. This depends on the stimulus leading to senescence and the cell type (Campisi & Fagagna, 2007). We used activation of SA‐β‐gal as a marker of senescence. This commonly used detection marker performs the hydrolysis of β‐galactosides into monosaccharides. To measure this reaction, the substrate X‐gal is hydrolyzed by SA‐β‐gal—which generates a blue color only in senescent cells. The assay is made more specific at pH = 6, which inhibits non‐senescence associated β‐gal. Whether this increased SA‐β‐gal activity in hyperoxia‐exposed cells has a physiological impact on cellular metabolism at pH 7 remains unclear.

Others have shown that serine 15 phosphorylation is important in p53‐induced senescence (Qian & Chen, 2013). We also preliminarily observed increased ser15 phosphorylation and senescence in mouse lungs after exposure to hyperoxia. (Peterson A, et al unpublished data, 2020). Overall, p53 regulation is extremely complex and may also involve other signaling events including phosphorylation at other sites, acetylation, and transcriptional pulse frequency (Hafner et al., 2019; Reed & Quelle, 2015). This has not been explored in this study. The mechanism by which p53 signaling leads to senescence in response to hyperoxia remains to be elucidated.

Hyperoxia also led to increases in glycolysis and compensatory glycolysis. This is in contrast to another study showing that glycolytic capacity decreases in MLE‐12 exposed to hyperoxia (Das, 2013), and that glycolysis does not change. The differences between our findings and theirs may be due to the specificity of the GRA we used, and the density of cells seeded in the Seahorse plate. In fact, in other studies (Scaffa A, et al unpublished data, 2020), we observed that MLE‐12 cells allowed to recover in room air after hyperoxia show decreased glycolysis and glycolytic capacity, which is in agreement with Das (2013). In hyperoxia, key glycolytic enzymes such as HK2 and TIGAR regulated glycolysis were inhibited by disruption of p53. Takebayashi et al. (2015) showed through metabolomics and transcriptomics that pRB upregulates glycolytic genes in oncogene‐induced senescence. In contrast, the pRBKO MLE‐12 cells did not show reduced glycolysis in hyperoxia suggesting a different mechanism in non‐cancer cells. We did not explore the role of Nrf2 in regulating glycolysis as has been suggested by others (Ohl et al., 2018). It has been shown that glycolysis regulation is cell cycle‐specific (Liu et al., 2020; Salazar‐Roa & Malumbres, 2017). Thus, it is possible that the higher level of glycolytic activity seen in our study is a reflection of where these cells arrest in the cell cycle in response to hyperoxia.

Others have shown that inhibiting glucose uptake can delay fibroblast senescence and that overexpression of glycolytic enzymes such as HK2 decreases oncogene‐induced senescence (Gitenay et al., 2014; Hariton et al., 2018). Surprisingly, we showed that p53KO MLE‐12 cells had increased glycolysis despite having less senescence. This may be attributable to the fact that p53 is a well‐known inhibitor of glycolytic activity in senescent cells (Wiley & Campisi, 2016). We suspect that p53 signaling is limiting glycolysis to suppress the exaggerated glycolytic response seen in senescent cells. Further confirmation is that p53 transcriptionally inhibited the glycolytic enzymes PFKFB3, and the rate limiting enzyme HK2. We noticed that although the absence of senescence leads to less cells that are highly glycolytic, overall glycolysis levels are highly upregulated because p53 cannot inhibit glycolysis, thereby resulting in its upregulation. Further studies are needed to understand how the p53 pathway and other signaling mechanisms are responsible for increased glycolysis in senescent cells. When fibroblasts undergo senescence, this results in a metabolic shift from oxidative phosphorylation to glycolysis (James et al., 2016; James et al., 2015). Whether this occurs in MLE‐12 is not clear and whether the increased glycolysis is a compensatory mechanism to maintain cellular energy supplies in the face of a hyperoxia‐mediated reduction in oxidative phosphorylation is also still unclear.

We showed that the senescent cells contributed to the majority of the increased glycolytic signal seen in hyperoxia. Glycolysis is an interesting therapeutic target for many disorders including cancer, where inhibition of glycolysis may lead to loss of ATP and cancer cell death (Zawacka‐Pankau et al., 2011). When glycolysis is disrupted, this leads to a switch from senescence to apoptosis in human lung cancer cells (Yao et al., 2017). This raises the possibility that altering glycolysis could prevent senescence and serve as a promising therapeutic strategy against CLD (Parikh et al., 2019). Our study is limited by the fact that we only used one immortalized MLE‐12 cell line, in which disrupting glycolysis did not diminish senescence. Whether inhibiting glycolysis would be useful to prevent senescence in primary lung epithelial cells exposed to hyperoxia or in mice remains to be determined. Senescence refers to a state of cell cycle arrest in which cells are not proliferative. Thus, follow‐up experiments to detect proliferation in cells exposed to hyperoxia and then allowed to recover in air may help to understand whether hyperoxia results in long term effects on senescence.

## 5 CONCLUSION

In this work, we aimed to study the relationship between p53 signaling, senescence, and glycolytic metabolism in response to hyperoxia in alveolar type II like lung cells. We showed that in MLE‐12 cells, exposure to hyperoxia leads to senescence, which is partially mediated by p53. Hyperoxia also leads to increased glycolysis, which is inhibited by p53. Even though p53 signaling leads to senescence and decreased glycolysis, the two events are independent of each other. Whether either senescence or altered metabolism contributes to alveolar simplification seen in CLD is being investigated in a mouse model. Since we show p53 activation in both mouse lungs and Type II cells exposed to hyperoxia, we speculate that therapeutic strategies modulating p53 and glycolysis may be useful to mitigate the detrimental consequences of hyperoxia in the neonatal lung. We do show that in response to hyperoxia, senescent cells are particularly glycolytic. Whether it would be beneficial to target this senescent cell population by reducing glycolysis to protect neonatal lungs exposed to hyperoxia is an important therapeutic consideration.

## CONFLICT OF INTEREST

The authors have declared that no conflict of interest exists.

## AUTHOR CONTRIBUTIONS

Conception and Design: AS, PAD; Data acquisition and analysis: AS, AP, JC, DG; Data Interpretation: AS, PAD; Drafting of the manuscript: AS, JC, PAD, HY; Revision of the manuscript: AS, PAD, HY.
